# Pleiotropy as the Mechanism for Evolving Novelty: Same Signal, Different Result

**DOI:** 10.3390/biology4020443

**Published:** 2015-06-19

**Authors:** John S. Torday

**Affiliations:** Harbor-UCLA Medical Center, 1124 West Carson Street, Torrance, CA 90502-2006, USA; E-Mail: jtorday@labiomed.org; Tel.: +1-310-222-8186

**Keywords:** pleiotropy, growth factor, growth factor receptor, cell-cell interaction, parathyroid hormone-related protein, prostaglandin E2, β-adrendergic receptor, type IV collagen, adipocyte differentiation related protein, neutral lipid trafficking

## Abstract

In contrast to the probabilistic way of thinking about pleiotropy as the random expression of a single gene that generates two or more distinct phenotypic traits, it is actually a deterministic consequence of the evolution of complex physiology from the unicellular state. Pleiotropic novelties emerge through recombinations and permutations of cell-cell signaling exercised during reproduction based on both past and present physical and physiologic conditions, in service to the future needs of the organism for its continued survival. Functional homologies ranging from the lung to the kidney, skin, brain, thyroid and pituitary exemplify the evolutionary mechanistic strategy of pleiotropy. The power of this perspective is exemplified by the resolution of evolutionary gradualism and punctuated equilibrium in much the same way that Niels Bohr resolved the paradoxical duality of light as Complementarity.

## 1. Pleiotropy, the Deus ex Machina (Ghost in the Machine)

Based on the conventional “snapshot” of an organism’s physiology, pleiotropy is generally construed as the same gene randomly utilized for various differing and flexible purposes. As a classic example of pleiotropy’s pervasive effects, the preeminent evolutionist George Williams utilized this phenomenon to explain, for example, that senescence occurs as the price for Darwinian reproductive advantage. He described this phenomenon as Antagonistic Pleiotropy [1]—when one gene controls more than one trait, one of these traits being beneficial to the organism’s fitness, and another detrimental to it.

However, might pleiotropy actually occur deterministically rather than by chance, based on specific physiologic principles, thereby revealing the true nature of evolution? It can be productively advanced that pleiotropy has fostered evolution through iterative interactions between the First Principles of Physiology and the ever-changing environment [2]. Pleiotropic novelties emerge through recombinations and permutations of cell-cell interactions for phenotypic adaptation based on both past and present conditions, in service to the future needs of the organism for its continued survival. Thus, in contrast to Antagonistic Pleiotropy based on descriptive biology, based on a cellular-molecular mechanistic approach senescence can be seen as the loss of cellular communication due to the natural decline in bioenergetics resulting from selection pressure for optimal reproductive success earlier in the life cycle of the organism [2].

## 2. Rubik’s Cube as a Metaphor for Pleiotropic Evolution

**Figure 1:**
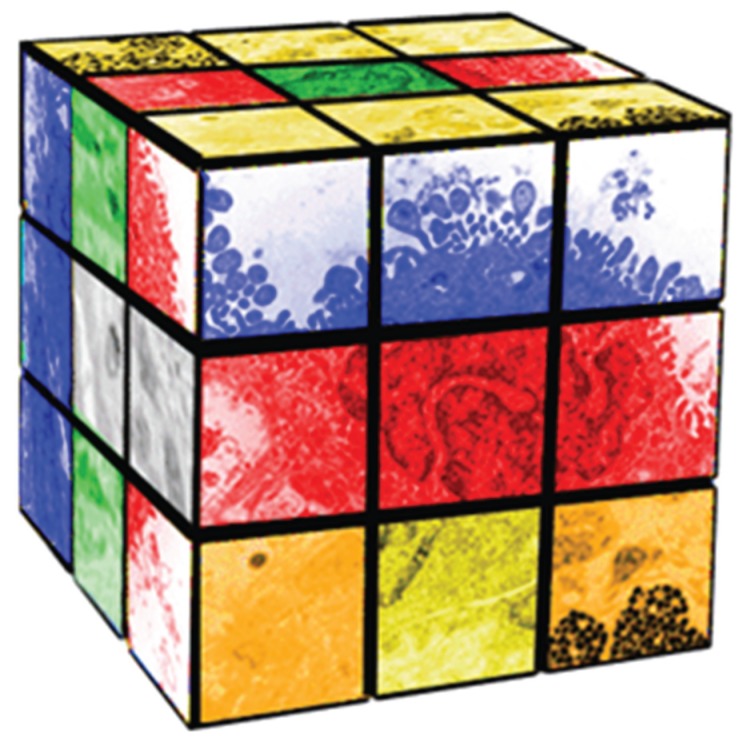
Pleiotropy as a Rubik Cube.

Erno Rubik invented his eponymous “cube” (Figure 1) to teach his students about spatial relationships and Group Theory [3]. By twisting and turning the cube, you can generate 4 × 10^19^ permutations and combinations of green, yellow, white, orange, red and blue squares in space and time. Similarly, as an embryo “twists and turns” in biologic space and time during development it generates hundreds of different cell-types via cell-cell signaling to form the human body; Lewis Wolpert, the renowned Developmental Biologist has said that “It is not birth, marriage, or death, but gastrulation, which is truly the most important time in your life”. That may be because it is at that stage in embryogenesis that the single layered cell membrane of the embryo becomes three-layered, generating the endoderm, mesoderm and ectoderm that ultimately give rise to the various cell-types of the organism [4,5]. Moreover, those various cell-types formulate tissue-specific homeostatic interactions to accommodate structure and function. The fact that the genes of each cell are all the same, and yet are all phenotypically different, both within and between tissues, has remained enigmatic within evolutionary development.

Pleiotropy is the expression of a single gene that generates two or more distinct phenotypic traits [6]—much like twisting a Rubik’s cube and forming various permutations and combinations of colors. In the case of the biologic process, it generates the various cellular phenotypes that compose the body, with equally varied homeostatic interactions [7]. If this process is followed phylogenetically and ontogenetically, it provides insights to the mechanisms of evolution [8], just as unicellular organisms gave rise to multicellular organisms under the iterative, interactive influences of both internal and external environmental selection pressures [9]. The Rubik’s cube metaphor for pleiotropy aids in understanding how one gene can affect multiple phenotypes. For example, it can be noted that there are images of cells on the faces of the Rubik’s cube in Figure 1 that are associated with different colors. As the cube is twisted to reconfigure the color combinations, those cellular images are re-permuted and recombined. The inference is that the cellular phenotypic traits are modified in much the same way as they would have been during the process of evolutionary adaptation. Therefore, the reallocation of genes and phenotypic traits is not due to random selection, but rather is determined by homeostatic constraints within each newly-established cellular niche [10]. Those constraints evolved from the unicellular bauplan and its homeostatic needs during the transition from one life cycle to the next, remaining consistent with those homeostatic constraints at every scale, phylogenetically, developmentally and physiologically alike [11]. If they do not, they can either be compensated for by other genetic motifs [12], they can be “silenced” [13], or they can be embryonically lethal [14]. It is this process that explains how and why physiologic traits are pleiotropically distributed throughout biologic systems. More importantly, it provides the mechanism for evolutionary novelty, since pleiotropy offers the opportunity to “repurpose” pre-existing genetic traits for different phenotypic functions, as needed.

In the book Evolutionary Biology, Cell-Cell Communication, and Complex Disease [9], the pleiotropic property of biology was utilized to explain the evolutionary mechanisms for both physiology and pathophysiology. In the former case, our work demonstrated how the alveolus of the lung and the glomerulus of the kidney are virtually the same functionally at the cell signaling level [9,15,16], even though they nominally seem to be structurally and functionally unrelated when seen from a descriptive perspective—one mediates gas exchange between the environment and the circulation, the other mediates fluid and electrolyte balance in the systemic circulation. However, both of these organs sense and transduce pressure signals, and thereby regulate homeostasis through stretch-regulation of Parathyroid Hormone-related Protein (PTHrP) production by the epithelium and its receptor-mediated signaling to specialized neighboring fibroblasts. Ironically, their homologous physiologic origins are recognizable through pathophysiologic conditions like Congestive Heart Failure and Goodpasture’s Syndrome. In the case of the former, heart failure commonly disrupts homeostatic control in both the lung and kidney [17] due to PTHrP dyshomeostasis in both [18,19]. In the case of the latter, the evolutionary adaptation to land mediated by the Goodpasture’s Syndrome Type IV collagen alpha3(IV)NC1 isomer, which is hydrophobic, protecting against water loss [19], can cause death due to the generation of autoantibodies that can cause heart and kidney failure [20].

Regarding the physiologic commonalities between the lung and kidney, in the case of the lung, the stretch-regulated PTHrP produced by the epithelial type II cell feeds-back to its receptor on the lipofibroblast to regulate lung surfactant production, reducing surface tension to maintain alveolar homeostasis [21]; in the case of the kidney, PTHrP produced by the epithelial podocytes that surround the fluid-filled space within the glomerulus regulate the mesangium, the thin mesodermal membrane supporting the glomerular capillary loops, homeostatically monitoring and regulating fluid and electrolyte balance in the systemic circulation [22].

## 3. The Lung as the Prototypical Pleiotropic Mechanism

The evolution of the lung was existential for the survival of land-dwelling vertebrates, since the rise in atmospheric temperature due to the Green House Effect of rising levels of carbon dioxide caused the drying up of bodies of water, forcing our forebears to adapt to land [23]. The physicochemically-integrated developmental and phylogenetic cell-cell interactions regulating lung surfactant offer the means of understanding the ontogenetic and phylogenetic structural-functional interrelationships at the cellular-molecular level between the decrease in alveolar diameter and the increased lung surface area for gas exchange. The counterbalancing of the otherwise pathological increase in alveolar surface tension due to the decrease in alveolar diameter would have resulted in its collapse, or atelectasis [24]; conversely, the evolution of epithelial-mesenchymal interactions for the concomitant thinning of the alveolar wall and the progressive efficiency of the surfactant system facilitated alveolar accommodation of gas exchange. This is the only biologic means for increasing oxygenation [25].

Beginning with the fish swim bladder as a biologic mechanism for adapting to water buoyancy—inflating to float, deflating to sink—fish have successfully exploited gas to optimize their adaptation to water buoyancy. All of the key molecular features of the mammalian lung as a reciprocating gas exchanger were already present in the fish swim bladder—surfactant phospholipid and protein to prevent the walls of the bladder form sticking together [26], PTHrP functioning during swim bladder development [27], and the β-Adrenergic Receptor regulating the filling and emptying of the swim bladder with gas absorbed from or secreted into the circulation [28]. These components of the evolutionary process were capable of re-permutation and recombination within the physiologic constraints of the existing structure and function to form the lung [29]. The only additional critical physiologic adaptation to be acquired was Neutral Lipid Trafficking (NLT) [30], mediated by Adipocyte Differentiation Related Protein (ADRP) [31], a member of the PAT (Perilipin, ADRP, TIP47) family of lipid transport and storage proteins wherever lipids are stored [32]. NLT likely evolved from the adaptive advantage of lipofibroblast neutral lipid storage, initially for protecting the lung gas exchange surface against oxidant injury [33], followed by its regulatory role as a means of more efficiently producing surfactant in response to the ever-increasing excursions of the alveolar wall in response to metabolic demand [30]. This is the epitome of the mechanism of pleiotropy, repurposing adipocyte metabolism for both the respiratory system and for the emergence of homeothermy [11], synergistically facilitating vertebrate adaptation to land through a common functional homolog.

## 4. The Lung as an Interactive Barrier: Homolog of the Plasma Membrane, Skin and Brain

Developmentally, the lung emerges from the foregut as an expansion of the surface of the alimentary tract [34]. As a homolog of the gut, the lung also acts as an interface between the internal and external environments of the body. However, the homology goes much deeper molecularly since the stratum corneum of the skin forms a lipid barrier on its surface much like the alveolar surfactant, forming tubular myelin as a membrane barrier [35]—in both cases [35,36], the epithelium secretes lamellar bodies composed of lipid-protein complexed with antimicrobial peptides. And the skin and brain are structurally-functionally homologous, both phylogenetically and pathophysiologically—the nervous system of the skin in worms gave rise to the central nervous system of vertebrates, referred to as the “skin-brain” [37]. Pathophysiologically, the skin and brain share common lipodystrophies in such neurodegenerative diseases as Niemann-Pick [38], Tay Sachs [39] and Gaucher’s Disease [40]. It has been speculated by some that this is a reflection of “too much of a ‘good thing’ going bad” [41]. In this case, the excessive myelination of axons in the brain causes tandem skin lipid lesions in association with brain neuronal pathology.

For example, the functional homology between the lung alveolus and kidney glomerulus are enacted by shared mechanotransducers for the physiologic stretching of their respective walls—in the case of the lung, alveolar PTHrP signals to increase surfactant production, preventing its collapse due to increased surface tension [42]. In the case of the kidney, the podocytes lining the glomerulus also secrete PTHrP, which then signals the mesangium to regulate water and electrolyte economy as a function of fluid distension [43]. In either case, the calcium-regulatory activity of PTHrP, which is ubiquitously expressed in all epithelial cells [44], has been embellished due to its myriad functionally evolved properties [45,46,47,48]. For example, due to its angiogenic property [48], PTHrP promotes microcirculatory capillary formation for gas exchange in the alveolar bed, and fluid and electrolytes in the glomeruli. Phylogenetically, within the fish kidney, the growth of the primitive filtering capillaries of the glomus would presumably have been stimulated locally by PTHrP, ultimately culminating in the expansion of the capillary network to form glomeruli, increasing the efficiency of water and electrolyte homeostasis in service to land adaptation [49].

## 5. NKX2.1, Thyroid, Pituitary and Lung Pleiotropy

The foregut is a plastic structure from which the thyroid, lung, and pituitary arise through the Nkx2.1/TTF-1 gene regulatory pathway [50]. Evolutionarily, this is consistent with the concept of terminal addition [51], since the deuterostome gut develops from the anus to the mouth [52]. Developmentally, when Nkx2.1/TTF-1 is deleted in embryonic mice, the thyroid, lung, and pituitary do not form during embryogenesis [53]. This provides direct experimental evidence for a genetic common denominator for all three organs. Their phylogenetic relationship has been traced back to amphioxus, and to cyclostomes, since the larval endostyle (a longitudinal ciliated groove on the ventral wall of the pharynx for gathering food particles) is the structural homolog of the adult thyroid gland [54].

## 6. The Phylogeny of the Thyroid

The endostyle is retained in post-metamorphic urochordates [55], and in adult amphioxus [56], but the post-metamorphic lamprey has a follicular thyroid gland, which is an evolved endostyle [57]. The presence of an endostyle in larval lampreys does not suggest direct descent of lampreys from protochordates, but rather that the evolutionary history of the lamprey is deep and ancient in origin, and that it shares the common feature of having a filter-feeding mechanism during its larval stage of development [58]. However, it is noteworthy that the other extant agnathan, the hagfish, possesses thyroid follicles before hatching [59]. Since hagfish evolution is considered to be conservative, going back 550 million years, this suggests that thyroid follicles could also be considered to have an ancient history [59].

## 7. An Evolutionary Vertical Integration of the Phylogeny and Ontogeny of the Thyroid

Mechanistically, the increased bacterial load consequent with the facilitation of feeding by the endostyle may have stimulated the cyclic AMP-dependent protein kinase A (PKA) pathway, since bacteria produce endotoxin, a potent PKA agonist [60,61]. This cascade may have evolved into regulation of the thyroid by Thyroid Stimulating Hormone (TSH), since TSH acts on the thyroid via the cAMP-dependent PKA signaling pathway [62]. This mechanism potentially generated novel structures such as the thyroid, lung, and pituitary, all of which are developmentally induced by the PKA-sensitive Nkx2.1/TTF-1 pathway [63]. The brain-lung-thyroid syndrome, in which infants with Nkx2.1/TTF-1 mutations develop hypotonia, hypothyroidism, and respiratory distress syndrome, or surfactant deficiency disease, provides further evidence for the coevolution of the lung, thyroid, and pituitary [64].

Developmentally, the thyroid evaginates from the foregut in the embryonic mouse beginning on day 8.5 [65], about one day before the lung and pituitary emerge, suggesting that the thyroid may have been a molecular prototype for the lung during evolution, providing a testable and refutable hypothesis. Adaptationally, the thyroid rendered molecular iodine in the environment bioavailable by binding it to threonine to synthesize thyroid hormone [66], whereas the lung made molecular oxygen tolerable, first by inducing fat cell-like lipofibroblasts as cytoprotectants [67], which then stimulated surfactant production by producing leptin [68], relieving the physiologic oxygenation constraint on the blood-gas barrier by making the alveoli more distensible [69]. This, in turn, would have further facilitated the use of rising oxygen in the atmosphere metabolically, placing further selection pressure on the alveoli, giving rise to the stretch-regulated surfactant system mediated by PTHrP and leptin [9]. Subsequent selection pressure on the cardiopulmonary system may have facilitated liver evolution, since the phylogenetically increasing size of the heart [70,71], accommodating the water-land transition, would have induced precocious liver development—induction of liver development is determined by the physical interaction between the heart and liver [72]—fostering increased glucose regulation, e.g., gluconeogenesis and glycogen storage/release. In turn, this may have fostered brain evolution since the brain is a glucose “sink” metabolically [73,74]. Further evolution of the brain, specifically the pituitary, would have served to foster the evolution of complex physiologic systems, culminating in endothermy/homeothermy in mammals and birds [11].

Both the thyroid and lung have played similar adaptive roles by accommodating otherwise toxic substances in the environment during vertebrate evolution. The thyroid has facilitated the utility of iodine ingested from the environment [75], whereas the lung has accommodated the rising oxygen levels during the Phanerozoic era [76]. Importantly, both the thyroid and lung have interacted synergistically in facilitating vertebrate evolution—for example, Thyroid Hormone stimulates embryonic lung morphogenesis during development [77], while also accommodating the increased lipid metabolism needed for surfactant production by driving fatty acids into muscle to increase motility [78], as opposed to maladaptively oxidizing circulating lipids to form toxic lipoperoxides [79]. The selection pressure for metabolism was clearly facilitated by the synergy between these foregut derivatives.

## 8. A Retrospective Understanding of Evolution

**Figure 2:**
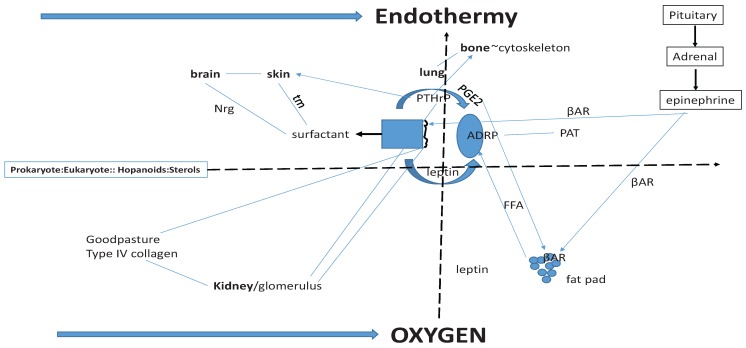
Origins of Vertebrate Physiologic Homologies. Sterols (far left) under the control of Hypoxia Inhibitory Factor-1 (Hif-1) link prokaryotes and eukaryotes together functionally; thus the major vertebrate physiologic homologies are linked through the induction of endothermy (top solid arrow) by atmospheric oxygen (bottom arrow). In the center of the Schematic is the molecular regulation of alveolar surfactant production. It is homologous with brain (neuregulin (NRG), and skin (tubular myelin (tm), both of which are under Parathyroid Hormone-related Protein (PTHrP) regulation. Lung and bone share functional homology through PTHrP stretch-regulated metabolism. Prostaglandin E2, leptin, Adipocyte Differentiation Related Protein (ADRP) and βAdrenergic Receptor (βAR) share homologies with fat pad Free Fatty Acid (FFA) regulation. The lung and kidney share functional homologies through PTHrP and Type IV Collagen (Goodpasture’s Syndrome). Periods of hypoxia due to lung evolution caused physiologic stress, stimulating the Pituitary-Adrenal Axis production of epinephrine, which both relieved the constraint on the alveoli by stimulating surfactant secretion, and stimulated peripheral fat cell secretion of FFAs, causing increased metabolic activity and body heat = endothermy.

Looking at the definitive structure and function of the mammalian alveolus (Figure 2), one can see the signature for phylogenetic traits that facilitated the evolution of land vertebrates from fish in a step-wise fashion. Referring to the Schematic (Figure 2), at the far left is the transition from prokaryotes to eukaryotes, which may have been the result of the effect of rising oxygen tension in the atmosphere on sterol production [80]. This scenario would resolve the age-old debate as to whether evolution was gradual or salutatory-it was both. This is a key insight to understanding mechanistic evolution. Historically, Darwin thought that evolution was a slow and gradual process [81]. He did not think that this process was smooth, but rather, that it should be presumed to be stepwise, with species evolving and accumulating through small variations over long periods of time. Darwin further speculated that if evolution were gradual that there would be fossil evidence for small incremental change within species. Yet Darwin and his supporters have been unable to find most of these hypothesized “missing links”. Darwin surmised that the lack of fossil evidence was due to the low likelihood that such critical transitions would have been preserved. Then, in 1972 [82] evolutionary biologists Stephen Jay Gould and Niles Eldredge suggested that the “gaps” in the fossil record were real, representing periods of stasis in morphology, calling this mode of evolution “punctuated equilibrium”. This infers that species are generally morpholgically stable, changing little for millions of years. This slow pace is “punctuated” by rapid bursts of change resulting in new species. According to this theory, changes leading to new species do not result from slow, incremental changes in the mainstream population. Instead, changes occur in populations living on the periphery, or in isolated populations where their gene pools vary more widely due to slightly different environmental conditions. When the environment changes, such peripheral or isolated species possess variations in morphology that might allow them an adaptive advantage.

A bridging concept can account for both Gradualism and Punctuated Equilibrium. The kinds of mechanisms that have been invoked for pleiotropy would account for both scenarios. As Darwin had surmised, evolution could have occurred on a continuous molecular basis microscopically in response to physiologic stress [83], occasionally leaving fossilized evidence once form and function reached a macro-scale, only making it seem as though evolution had occurred in bursts (yet the molecular evidence can be seen in the continuum from ontogeny and phylogeny to pathophysiology!).

A scenario for two differing rates of evolutionary change is all the more cogent when one superimposes the episodic increases and decreases in atmospheric oxygen that have been documented over the last 500 million years, referred to as the Berner Hypothesis [76]. Within this theory, the increases in atmospheric oxygen caused the well-documented increases in the size of land animals [83]. However, the decreases have never been considered before, yet would predictably have had profound effects on vertebrae evolution, given that hypoxia is the most potent effector of complex physiologic systems [84]. Elsewhere [11], a novel mechanism for the evolution of endothermy/homeothermy based on the interactions between the pulmonary and neuroendocrine/endocrine systems has been evoked that allows for the arc of the Cambrian Burst, culminating in the crown species of mammals and birds. This perspective is validated by the pleiotropic effects of the specific gene duplications for the PTHrP Receptor [85], the β-Adrenergic Receptor [86], as well as the differentiation of the Glucocorticoid Receptor [87] and the evolution of the Goodpasture’s Syndrome Type IV collagen isomer [19], all of which occurred during the water-land transition. These events corroborate the repurposing of pre-existing genes for novel phenotypic adaptations.

Even earlier in vertebrate evolution, sterols may have liquified the bacterial cell wall [80], possibly due to rising levels of oxygen in the atmosphere [88] stimulating sterol production. That event would have marked the phenotypic transition from prokaryotes to eukaryotes, the former having hard exterior walls, the latter having compliant cell membranes. That transition may have been further catalyzed by the nascent synthesis of cholesterol [89], under positive control by Hypoxia Inducible Factor-1 [90], catalyzing the evolution of eukaryotes (see Figure 2). The two horizontal, bolded arrows for “endothermy” and “oxygen” were the major drivers of vertebrate evolution. All three of highlighted processes—prokaryote/eukaryote evolution, oxygen and endothermy—have acted synergistically to promote vertebrate evolution, indicated by the dotted arrows that interconnect them.

## 9. Denouement

The seemingly serendipitous occurrences of pleiotropy based on the conventionally descriptive understanding of biology are over-arched by the synchronically mechanistic basis for pleiotropy, emanating from the cell-cell signaling principles elucidated above. Thus, the deep, otherwise-unobvious pleiotropic homologies transcend the superficialities of comparative anatomy, only being revealed by knowledge of molecular developmental and phylogenetic physiologic motifs ([91], The deepest of these are related to the physiologic effects of stretching, or mechanotransduction, on surfactant metabolism, which refers all the way back to biologic adaptation to gravitational force, the most ancient, omnipresent and constant of all environmental effectors of evolution [92,93].

For example, the alveolar type II (ATII) cells produce Prostaglandin E2 (PGE2) [94], particularly when they are distended [95], causing secretion of lipid substrate from lipofibroblasts for lung surfactant phospholipid production by the ATII cells [96]; without PGE2, the lipids would remain bound within the lipofibroblasts. This effect of PGE2 on the secretion of Free Fatty Acids (FFAs) from lipofibroblasts is homologous with the release of FFAs from peripheral fat cells, a trait that hypothetically evolved as a consequence of the evolution of endothermy [11]. To alleviate the periodic hypoxic constraints on the evolving alveolar bed, stress induced adrenalin stimulated surfactant secretion to increase gas exchange transiently until the indigenous PTHrP mechanism could generate more alveoli [96]. Thus, the pleiotropic co-evolution of the PGE2 mechanism facilitating FFA utilization in both the lung and fat pad was not a chance event; it was synergistic when viewed within the context of the evolving lung’s effect on endothermy [11]. In further support of this hypothesis, the role of the lung in the evolution of endothermy is further evidence for the causal evolutionary interrelationship between the pulmonary and neuroendocrine systems, both mediated by PTHrP signaling [97,98]. Yet again, this is not a chance event; periods of hypoxia due to the continuous evolution of the lung would have caused physiologic stress, stimulating adrenalin production by the adrenal medulla. Adrenalin production would have had the dual adaptive benefit of increasing alveolar oxygenation [99], and releasing FFAs from the peripheral fat pads [100]. The release of excess FFAs from the fat pad would otherwise have been toxic [101], but instead adaptively increased body temperature [11], complementing the evolution of dipalmitoylphosphatidylcholine, the surface-active phospholipid in mammalian alveoli, which is 300 percent more surface-active at 37 °C than at 25 °C [102].

A similar physiologic evolutionary interrelationship emerges from the etiology of Goodpasture’s Syndrome. The disease is caused by an autoimmune reaction to an evolved isoform of Type IV collagen [103]. Alpha 3(IV)NC1 Type IV collagen is absent from worms and flies, but appears in fish [19]. However, it does not generate the pathogenic Goodpasture’s Syndrome antibody [19]. It is ubiquitous in amphibians, reptiles, birds and mammals. It has the evolutionarily-relevant physicochemical characteristic of being more hydrophobic than other Type IV collagens, offering a functional role in preventing water loss across the lung and kidney epithelia in adaptation to land. The fact that this specific Type IV collagen isoform evolved during the process of land adaptation is unlikely to have occurred merely by chance, given its ability to prevent water loss on land [19].

Thus, not unlike Chemistry and Physics, Biology, is also founded on First Principles that can be understood ontologically and epistemologically rather than through dogmatic teleologic mechanisms and tautologic concepts [104]. George Williams’ Antagonistic Pleiotropy hypothesis for senescence was alluded to above—in large part, this perspective is reflective of the systematic error authored by Ernst Mayr [105] that there are proximate and ultimate mechanisms of evolution that must be dissociated from one another based on Darwinian principles of mutation and selection. However, that dictum was formulated more than sixty years ago. Theorists that offered differing perspectives, such as Haeckel, Spemann and Lamarck have generally been dismissed. However, in the interim a great deal more about biology has been learned that re-energizes some previously disregarded principles towards understanding evolutionary development. This is particularly true within cell biology, where pathways can be identified that inform us that there is a continuum between the proximate and ultimate mechanisms of evolution—Mayr exemplified this principle using bird migration, which was then too complex to be understood as one continuous process, yet we now know how ambient light affects the neuroendocrine system to foster migratory behavior.

As an extension of the insights gained by seeing pleiotropy through the lens of mechanistic pleiotropy, repurposing of the same genetic signaling cascade to form novel phenotypes, heterochrony can be seen in the same way—the mechanism of heterochrony has never been provided before, it has only been described [106]. Haeckel described the concept of Heterochrony as a way of expressing how development could facilitate evolutionary change [107]. To this day, no one has expressed heterochrony as a mechanism for reallocating cell-cell signaling to accommodate adaptive change, yet it is the premise we have used throughout this book.

## 10. Conclusions

It was Thomas Kuhn, the author of The Structure of Scientific Revolutions [108], who said that an indicator of a paradigm shift was a change in the language—going from a descriptive to a mechanistic way of thinking about pleiotropy and heterochrony would reflect such a paradigm shift.

Indeed, Haeckel, Spemann and Lamarck had many correct surmises about the mechanistic biologic principles that they each addressed—recapitulation theory, the embryologic “organizer”, and acquired characteristics. In their time, they lacked the technical ability to support their hypotheses. However, the novel perspective on pleiotropy expressed herein honors both old concepts and new. Our own evolving understanding of evolutionary mechanisms generates a compelling narrative for evolution as a continnum of physiologic adaptations towards rewarding homeostatic mechanisms that permit cells to thrive in diverse environments.

Cells solve problems—they use the tools that they have or can generate [109]. Many generations of scientists have attempted to discern the puzzle of evolutionary development, yet they have lacked the tools that can be productively employed today. What we have now learned is in many ways unexpected. Contrary to our expectation, what was old can again become new. In that sense, this paper is dedicated to those who have labored before us. Their efforts can now be married to compelling research. Through this combination, a new paradigm for evolutionary development unfurls that is congruent with the dominant truth that can be asserted about our physiologic path from First Principles. It is clearly evident that all complex organisms unavoidably must return to their unicellular roots [10,11]. The physiological pathways and the cellular communication mechanisms that underscore it explain the imperative for this immutable recapitulation.

The resolution of the evolutionary significance of pleiotropy is tantamount to Niels Bohr’s eloquent explanation for how light could be both wave and particle based on principles of Quantum Mechanics. In his Complementarity lecture at Lake Como, Switzerland in 1927 he resolved this paradoxical duality by explaining that it was an artifact of the way in which the light was measured (Bohr Como Lecture) [110]. Similarly, the cell is both genetic and phenotypic, depending upon the metric, yet in reality it is integral whole whose fate is determined by the ever-transcendent mechanisms that perpetuate it [11]. In his groundbreaking book Wholeness and the Implicate Order the physicist David Bohm [111] explains how our subjective senses cloud our perception of reality. As in Physics, recognizing this dichotomy is key to future progress in biology and medicine.
